# Trends in seasonal warm anomalies across the contiguous United States: Contributions from natural climate variability

**DOI:** 10.1038/s41598-018-21817-9

**Published:** 2018-02-21

**Authors:** Lejiang Yu, Shiyuan Zhong, Warren E. Heilman, Xindi Bian

**Affiliations:** 1 SOA Key Laboratory for Polar Science, Polar Research Institute of China, Shanghai, China; 2 Department of Geography, Environment and Spatial Sciences, Michigan State University, East Lansing, MI USA; 3 Northern Research Station, US Forest Service, Lansing, Michigan USA

## Abstract

Many studies have shown the importance of anthropogenic greenhouse gas emissions in contributing to observed upward trends in the occurrences of temperature extremes over the U.S. However, few studies have investigated the contributions of internal variability in the climate system to these observed trends. Here we use daily maximum temperature time series from the North American Land Data Assimilation System Phase 2 (NLDAS-2) dataset to identify trends in seasonal warm anomalies over the contiguous U.S. in the three most recent decades and explore their relationships to low-frequency modes of internal climate variability. The results reveal substantial upward trends in the frequency of warm anomalies in all seasons and in all regions of the U.S., except for portions of the Intermountain West in winter where significant downward trends occur. The strengths and regional coverage of the trends, however, differ considerably by season. These trends can be explained, in part, by the large-scale anomalous atmospheric circulations associated with low-frequency sea-surface temperature oscillations characterized by the Pacific Decadal Oscillation (PDO) and the Atlantic Multidecadal Oscillation (AMO). The association between the upward trends in the seasonal warm anomalies and PDO and AMO is further confirmed by the century-long (1871–2012) Twentieth Century Reanalysis dataset.

## Introduction

Since about 1970, mean surface temperatures have been on a rising trend across the U.S., with most of the warming occurring over the West, the northern Plains, and the Northeast, and little change to slight cooling in the Southeast^1,2^. Compared to mean temperatures, temperature extremes have greater social, ecological, and economic consequences^3^. For example, a heat wave in early July of 1993 resulted in 111 deaths in Philadelphia and severe agricultural losses in the Delmarva Peninsula poultry industry^4^. Frequent heat waves often lead to extremely low dead fuel moisture content and increasing the potential for large wildfires following ignition events^5^. Hence, it is essential to understand the trends in warm extremes and the underline mechanisms so that better seasonal forecasting of extreme temperature events can be achieved.

Numerous studies have investigated temperature extremes over the U.S using climate data^6–13^. Both upward and downward trends have been identified for extreme maximum temperatures, depending on the region and time period studied. On the century time scale and averaging across the U.S., these studies suggest that there has been a slight downward trend in extreme maximum temperatures over the last century, due to the high frequency of occurrences of warm extremes during the drought periods in the early part of the 20th century. But in recent decades, the trends are predominantly upward in most regions of the U.S. These climatological studies, however, stopped short of offering any explanation for the observed trends.

Several studies on climate-change effects have attributed the changing frequency and intensity of maximum temperature extremes over the 20^th^ century to anthropogenic forcing through processes such as snow albedo and surface moisture feedbacks^14–20^. In addition to anthropogenic forcing of the climate system, natural internal variability in the climate system, such as the Arctic Oscillation (AO) and the North Atlantic Oscillation (NAO), has also been indicated as contributors to changes in the occurrence of warm temperature extremes^21^.

In this study, we further examine the trends in seasonal warm extremes defined as daily maximum temperature values greater than the 95^th^ percentile for the season in the contiguous U.S., with a focus on understanding to what extent and through what mechanisms these trends can be accounted for by low-frequency modes of internal climate system variability. We utilize time series of daily maximum temperatures from two reanalysis datasets: the North American Land Data Assimilation System Phase 2 (NLDAS-2) at 1/8 degree horizontal spatial resolution covering the 1979–2014 period^22^ and the Twentieth Century Reanalysis at 2.5 degree horizontal spatial resolution spanning the 1871–2012 period^23^. Sea surface temperature data and various climate indices are also utilized in the analyses. Because gridded reanalysis data have limited ability to capture the true extreme values due to their relatively low spatial and temporal resolution (Fig. S1), we will use seasonal warm anomalies instead of seasonal warm extremes henceforward.

## Results

**Figure 1:**
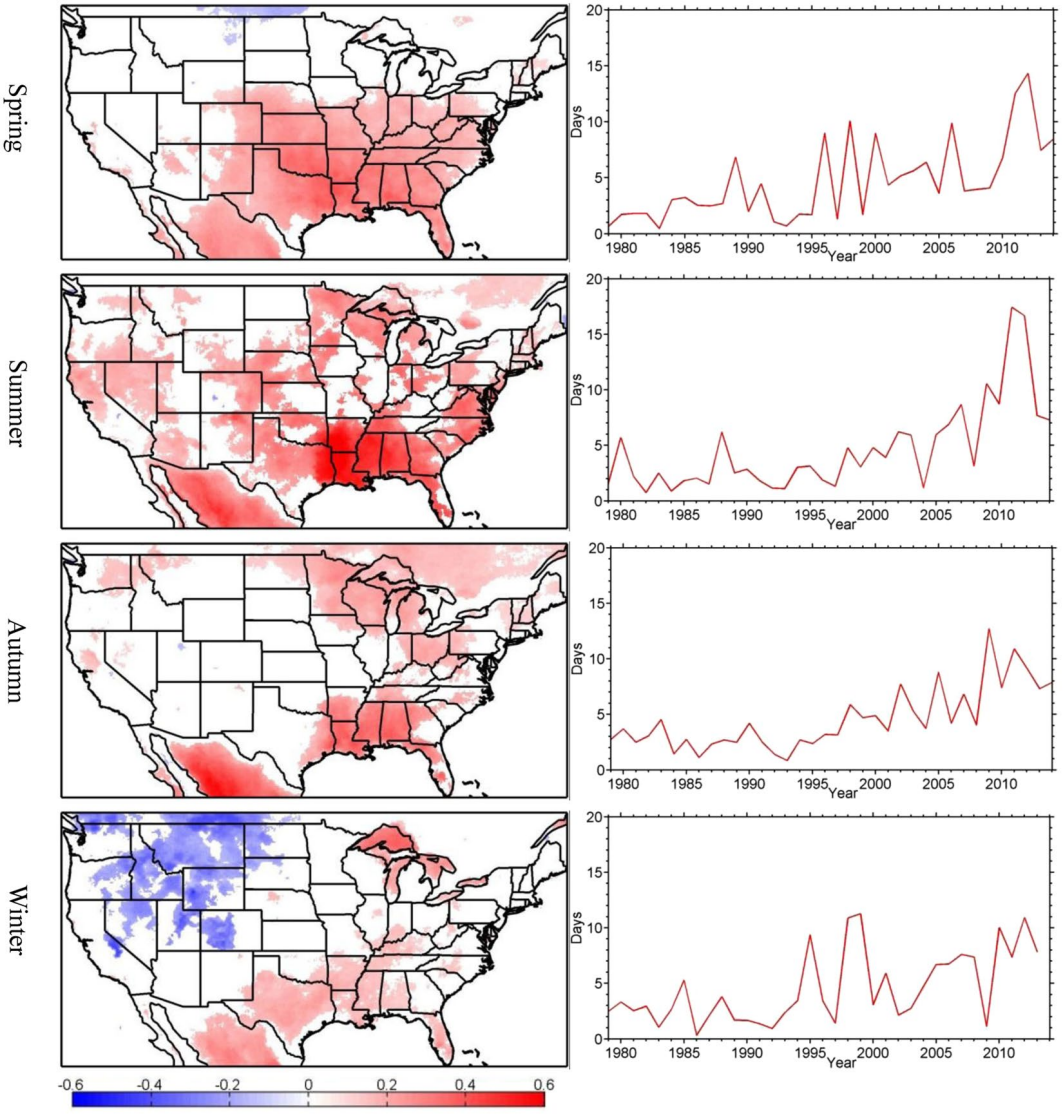
Spatial patterns (left column) and time series (right column) of the number of seasonal warm anomalies derived from NLDAS-2 data. Only significant trends above the 95% confidence level are shown (unit: day yr^−1^) and the time series is averaged over all grid points with statistically significant positive trends. This figure is created using MATLAB & Simulink Release 2010b (www.mathworks.com).

Over the past three decades, the occurrences of seasonal warm anomalies have generally been on an increasing trend across the U.S., except for parts of the Intermountain West and the western Northern Plains in winter where a decreasing trend has occurred (Fig. 1). This is not surprising given the general warming trends in these decades. The rates of change, however, show large spatial and seasonal variations. In summer, significant increasing trends characterize much of the U.S., with the largest trend of 0.62 days year^−1^ found over the Southeast. In spring, significant upward trends are found over the southeastern, south-central and lower mid-western portions of the U.S. Rising trends in autumn characterize the Southeast and Upper Midwest. Winter is the only season when a significant decreasing trend is found in areas of the Intermountain West and the western edge of the Northern Plains, while increasing trends remain in parts of the southeastern and south-central U.S.

Averaging over all grid points with significant upward trends, the mean rates of increase in the number of seasonal warm anomalies are 0.22, 0.26, 0.20 and 0.18 day yr^−1^ for spring, summer, autumn and winter, respectively. In winter, the average rate over the areas of downward trends (−0.24 day yr^−1^) exceeds that of the upward trends (0.18 day yr^−1^). For all seasons, a pronounced shift in the rate of change occurs in the late 1990’s. For example, the number of springtime warm anomalies is 2.29 when averaged over the period of 1979–1995, but 7.03 when averaged over the period of 2000–2014. Similar differences between the two periods exist for the other seasons. This shift in the late 1990’s appears to dominate the trends.

**Figure 2:**
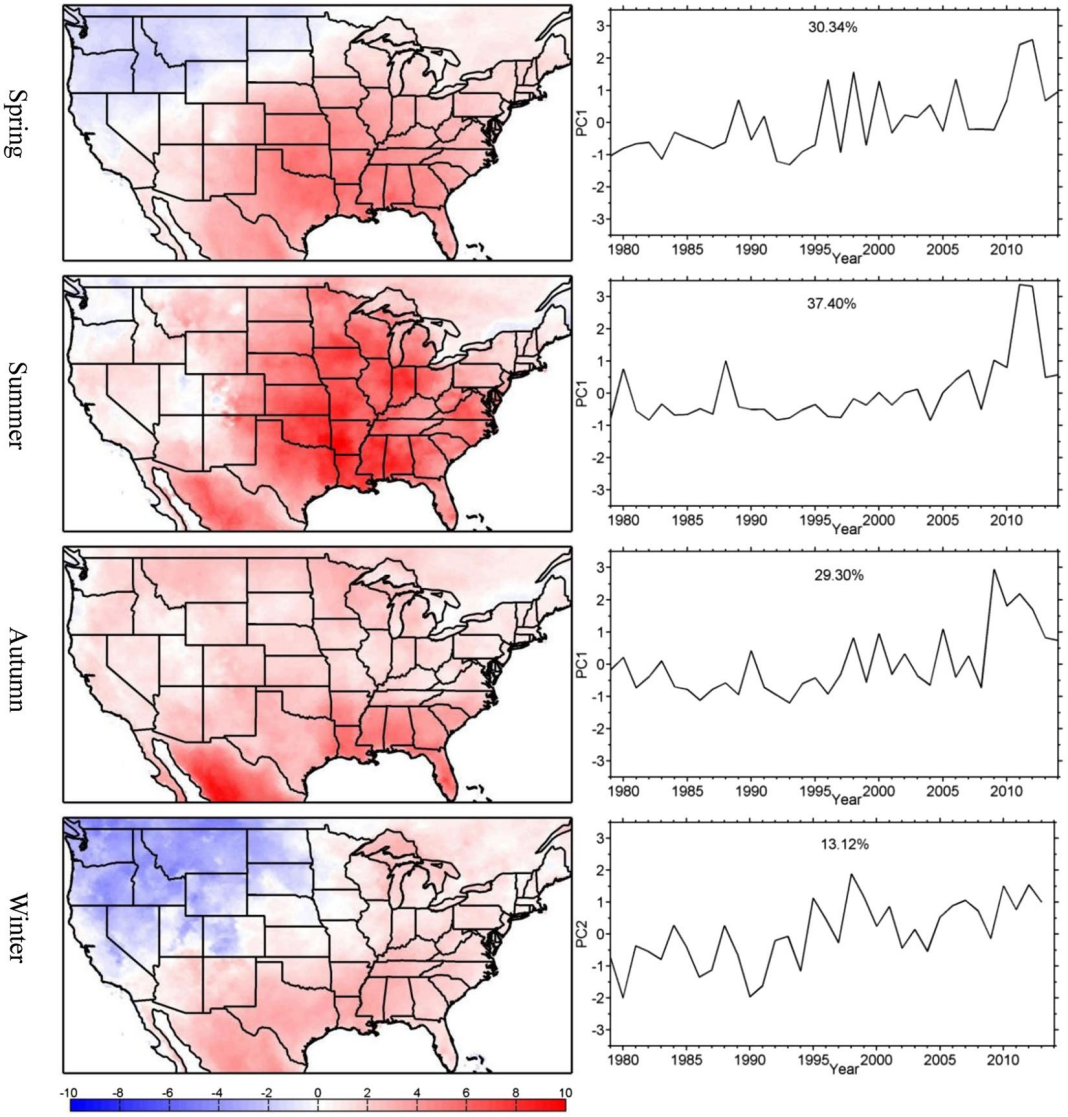
EOF Spatial patterns (left column) and time coefficients or PCs (right column) for the number of seasonal warm anomalies (unit: day yr^−1^) derived from NLDAS-2 data. EOF2 is shown for the winter season and EOF1 is shown for the other seasons. This figure is created using MATLAB & Simulink Release 2010b (www.mathworks.com).

To explain the trends, empirical-orthogonal-function (EOF) analyses were applied to the anomalies of the number of seasonal warm anomalies relative to the 1979–2014 climatology. Figure 2 shows the spatial patterns and time coefficients (principal components (PCs)) of the first modes (EOF1 and PC1) for the spring, summer and autumn seasons. The second mode is shown for winter because the wintertime EOF1 shows a uniform distribution across the U.S., and PC1 shows no significant trend.

Similar to the trends shown in Fig. 1, EOF1 values for the summer and autumn seasons are nearly all positive, indicating more frequent occurrences of seasonal warm anomalies (Fig. 2). The springtime EOF1 and wintertime EOF2 show negative trends in the Northwest and parts of the Northern Plains and positive trends in the other regions (Fig. 2). Despite the relatively large interannual variability, the PC time series show significant upward trends, with a switch from mainly negative values prior to the late 1990’s to mainly positive values thereafter (Fig. 2). The correlation coefficients between the PCs (Fig. 2) and the spatially averaged trends (Fig. 1) are greater than 0.8 for all seasons.

**Figure 3:**
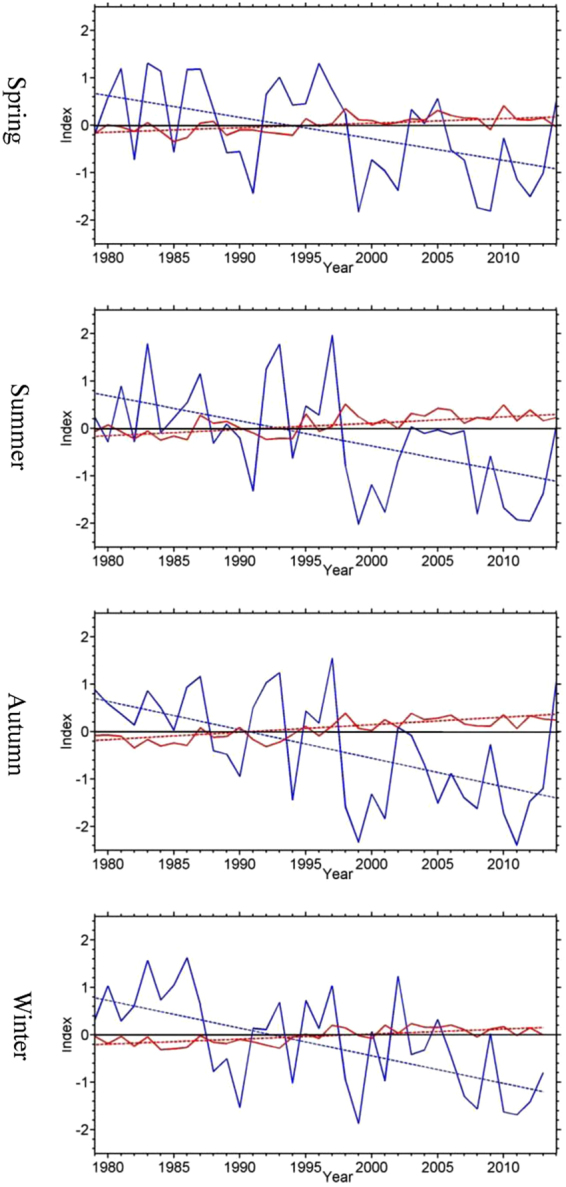
Time series of the seasonal PDO (solid blue lines) and AMO (solid red solid lines) indices and their trends (dashed lines). Zero is indicate by the solid black line. This figure is created using MATLAB & Simulink Release 2010b (www.mathworks.com).

At inter-decadal timescales, the Pacific Decadal Oscillation (PDO)^24^ and the Atlantic Multidecadal Oscillation (AMO)^25^ are major modes of climate variability in the North Pacific and Atlantic Oceans. The seasonal PDO and AMO indices show a phase shift in the mid-1990s (Fig. 3), with a change from mostly positive to negative values for PDO and vice versa for AMO. The seasonal AMO indices exhibit a significant upward trend, similar to the trend in PCs, while the PDO indices have a significant downward trend (Fig. 3 and Table 1). There are significant (above the 95% confidence level) correlations between the PCs and the PDO and AMO indices (Tables 2 and 3). Previous studies have also identified a relationship between extreme temperatures in the U.S. and changes in SSTs characterized by the PDO^26^.

**Table 1:** Trends in the seasonal PDO and AMO indices (unit: yr^−1^). All the trends are significant at the 95% confidence level.

	Spring	Summer	Autumn	Winter
PDO	−0.05	−0.05	−0.06	−0.06
AMO	0.01	0.01	0.02	0.01

**Table 2:** Correlations between the PCs for seasonal warm anomalies and the PDO index for the same and previous seasons. All the correlations are significant at the 95% confidence level with the exception of Winter PC2 and Spring PDO index.

PDO index	Spring(PC1)	Summer (PC1)	Autumn (PC1)	Winter (PC2)
spring	−0.36	−0.38	−0.42	−0.26
summer	−0.36	−0.50	−0.46	−0.42
autumn	−0.48	−0.47	−0.45	−0.58
winter	−0.41	−0.45	−0.52	−0.50

**Table 3:** Correlations between the PCs for seasonal warm anomalies and the AMO index for the same and previous seasons. All the correlations are significant at the 95% confidence level.

AMO index	Spring (PC1)	Summer (PC1)	Autumn (PC1)	Winter (PC2)
spring	0.44	0.38	0.46	0.66
summer	0.58	0.43	0.50	0.60
autumn	0.56	0.43	0.52	0.56
winter	0.53	0.36	0.41	0.49

**Figure 4:**
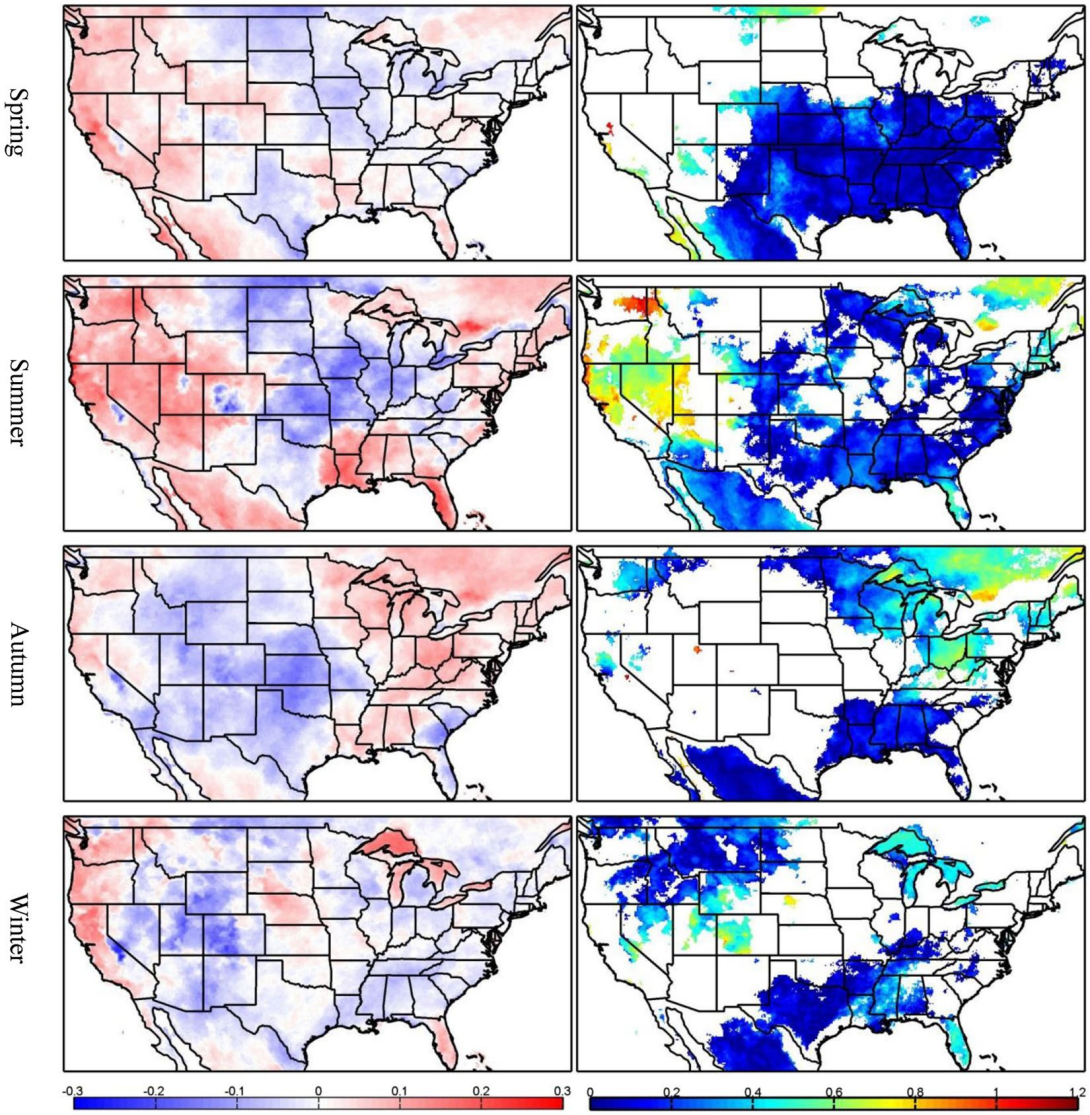
Residual trends (left column) and the ratios of the residual trends to the total trends (right column) (unit: day yr^−1^) for the number of seasonal warm anomalies derived from NLAS-2 data. This figure is created using MATLAB & Simulink Release 2010b (www.mathworks.com).

The amount of trends explained by the PCs can be measured by the ratios of the residual trends to the total trends, where the residual trends are calculated by subtracting the trends in the PCs (as shown in Fig. 2) from the total trends (as shown in Fig. 1). Although there is considerable variation across the U.S., the magnitudes of the residual trends are smaller than 0.3 day yr^−1^. The ratios of the residual trends to the total trends are generally less than 20%, with large values over the western U.S. and southeastern Canada during summer, and over the northeastern U.S. during autumn (Fig. 4). The large ratios in some regions are due to small total trends in the denominator (<0.2 day yr^−1^). The average ratios over all grid points with statistically significant trends are 25, 35, 30, and 22% for spring, summer, autumn and winter, respectively. The relatively small ratios indicate that the trends in the seasonal warm anomalies can be largely explained by the trends in the PCs.

**Figure 5:**
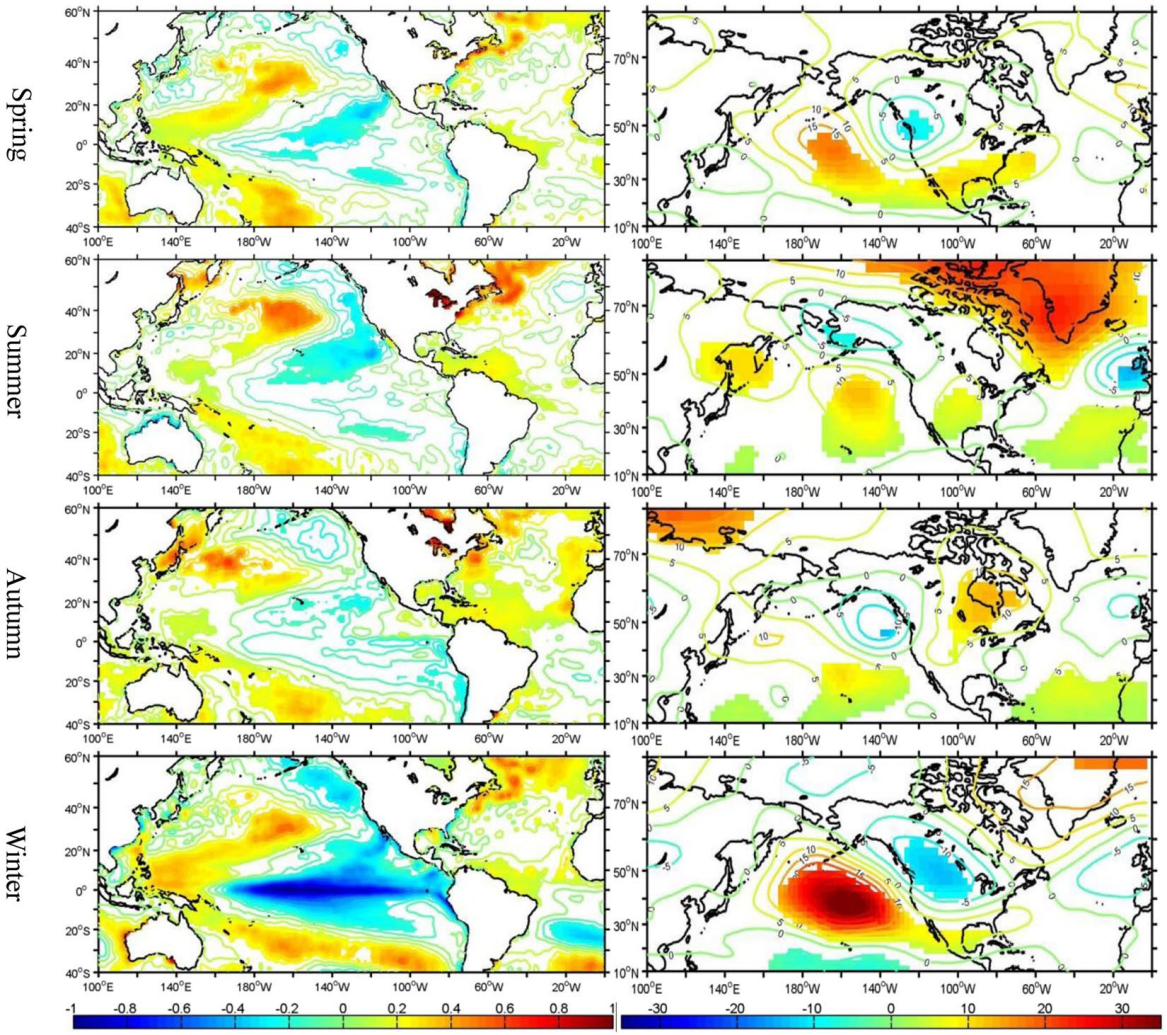
The anomalous sea surface temperature (SST) (°C) (left column) and 500-hPa geopotential height (gpm) (right column) regressed to the PCs of the number of seasonal warm anomalies for the period 1979–2014. The filled regions are significant at the 95% confidence level. The SST contour interval is 0.1 °C. This figure is created using MATLAB & Simulink Release 2010b (www.mathworks.com).

**Figure 6:**
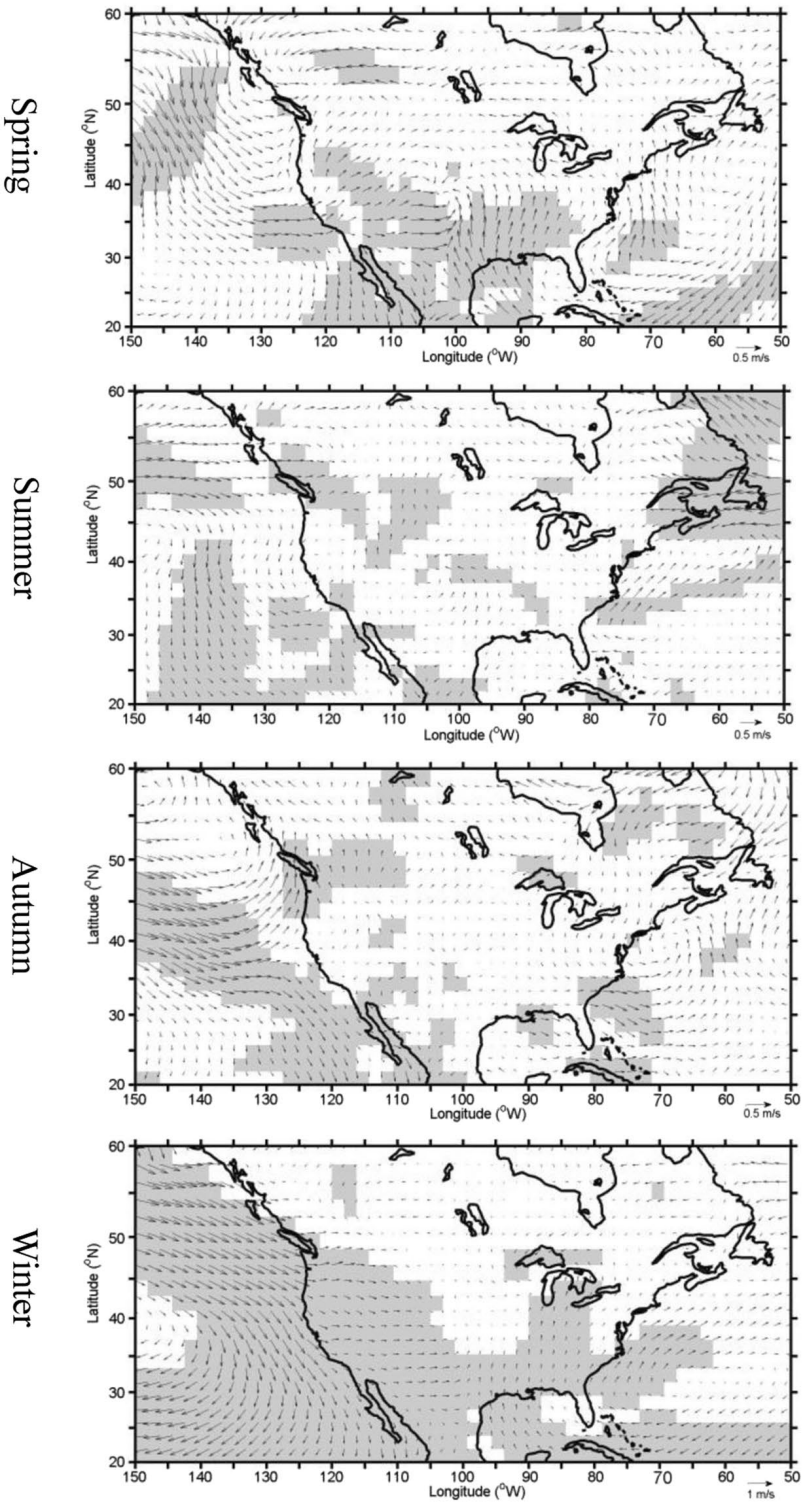
The same as Fig. 5, but for the 10-m anomalous wind vectors. The shading identifies those areas where the wind anomalies are statistically significant at above 95% confidence level. This figure is created using MATLAB & Simulink Release 2010b (www.mathworks.com).

To understand the trends explained by the PCs in the context of atmospheric circulation anomalies, regression analyses were carried out, where the SSTs, 500-hPa geopotential heights, and 10-m wind vectors were regressed to the PCs (Figs 5 and 6). The SST regression maps show a spatial pattern indicative of the negative phase of the PDO over the North Pacific Ocean and the positive phase of the AMO over the North Atlantic Ocean (Fig. 5), which is consistent with the significant correlations between the PCs and the PDO and AMO indices (Tables 2 and 3). In addition, the spatial SST regression pattern over the tropical Pacific Ocean resembles the La Niña Modoki pattern, particularly in winter. The regression patterns of the anomalous 500-hPa geopotential heights in spring and winter (Fig. 5) resemble the negative Pacific North America (PNA) teleconnnection pattern, with negative height anomalies over western North America favoring cold air intrusions and fewer opportunities for warm anomalies. The anomalous northwesterly winds over the northwestern U.S. also contribute to lower seasonal temperatures and reduce the occurrences of warm extremes in this region (Fig. 6). The opposite occurs over the eastern U.S. This is consistent with the result from a previous study^27^ that linked La Niña Modoki to the wintertime warm (cold) anomalies over the southeastern (northwestern) U.S. In summer and autumn, most of the country is dominated by positive height anomalies (Fig. 5), which, according to the strong association between anti-cyclonic anomalies and extreme heat over North America^28^, would favor warm anomalies in these seasons. In addition, the low wind speeds across the U.S. (Fig. 6) can be conducive to more extreme warming episodes.

**Figure 7:**
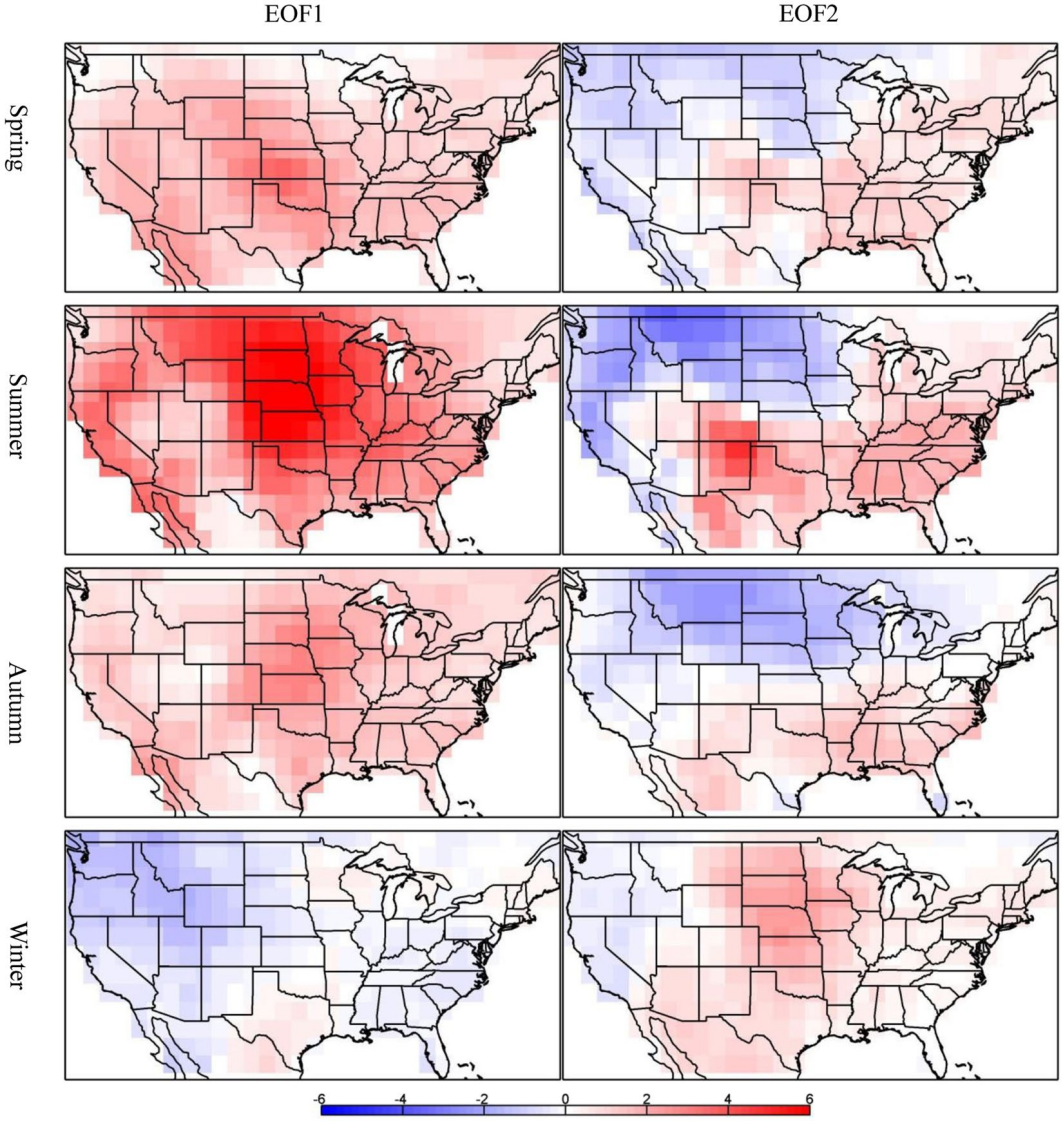
The spatial patterns of the first (left column) and second (right column) EOF modes for the seasonal warm anomalies (unit: day yr^−1^) from the Twentieth Century Reanalysis. This figure is created using MATLAB & Simulink Release 2010b (www. mathworks.com).

**Figure 8:**
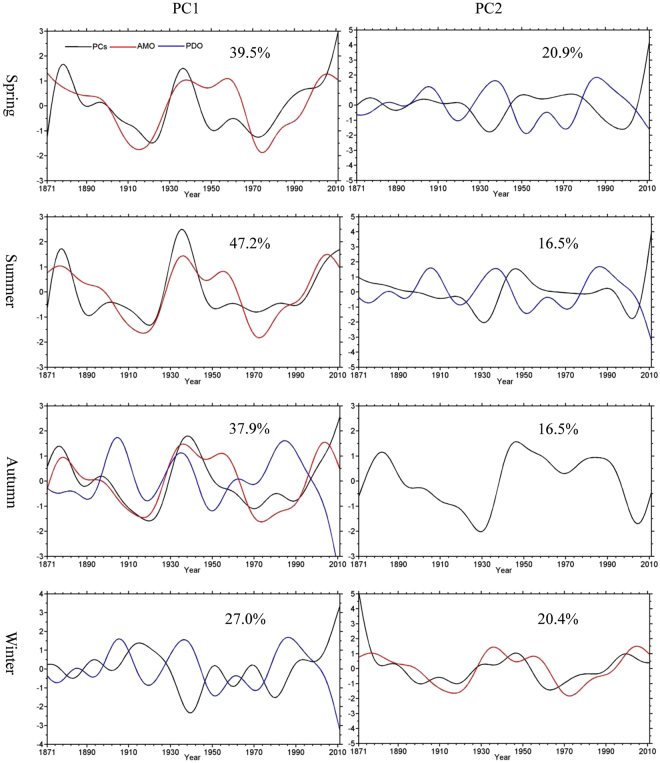
The time coefficients (PCs) of the first (left column) and second (right column) EOF modes for the seasonal warm anomalies (unit: day yr^−1^). The black, red, and blue lines indicate PCs, the AMO index, and the PDO index, respectively. Only the AMO and PDO indices that have statistically significant correlations (95% confidence level) with PCs are shown. The number in each plot denotes the percentage of the total variance explained by the modes. This figure is created using MATLAB & Simulink Release 2010b (www.mathworks.com).

The relationships between the number of seasonal warm anomalies and the PDO and AMO, which were based on relatively short time series (1979–2014) with limited utility for dealing with climate variability at decadal and multi-decadal time scales, were confirmed by repeating the above analyses using the century-long (1871–2012) Twentieth Century Reanalysis dataset. The results (Figs 7 and 8) show strong positive correlations between PC1 and AMO in the spring (0.63) and summer (0.75). However, PC2 is more negatively related to the PDO in the spring (−0.65) and summer (−0.47). In winter, PC1 is negatively correlated with the PDO (−0.61) while PC2 is positively correlated with the AMO (0.44). For these three seasons, the two leading EOF modes explain nearly 50% of the total variances, and the sums of their spatial patterns are similar to the spatial patterns derived from the shorter time series shown in Fig. 3. For autumn, the first mode, which explains 38% of the variance, is correlated much more strongly to the AMO (0.82) than to the PDO (−0.34). In summary, these results based on long-term data further support the connection between the decadal and multi-decadal variations of seasonal warm anomalies and low-frequency SST oscillations characterized by the AMO over the North Atlantic and the PDO over the North Pacific Ocean.

**Figure 9:**
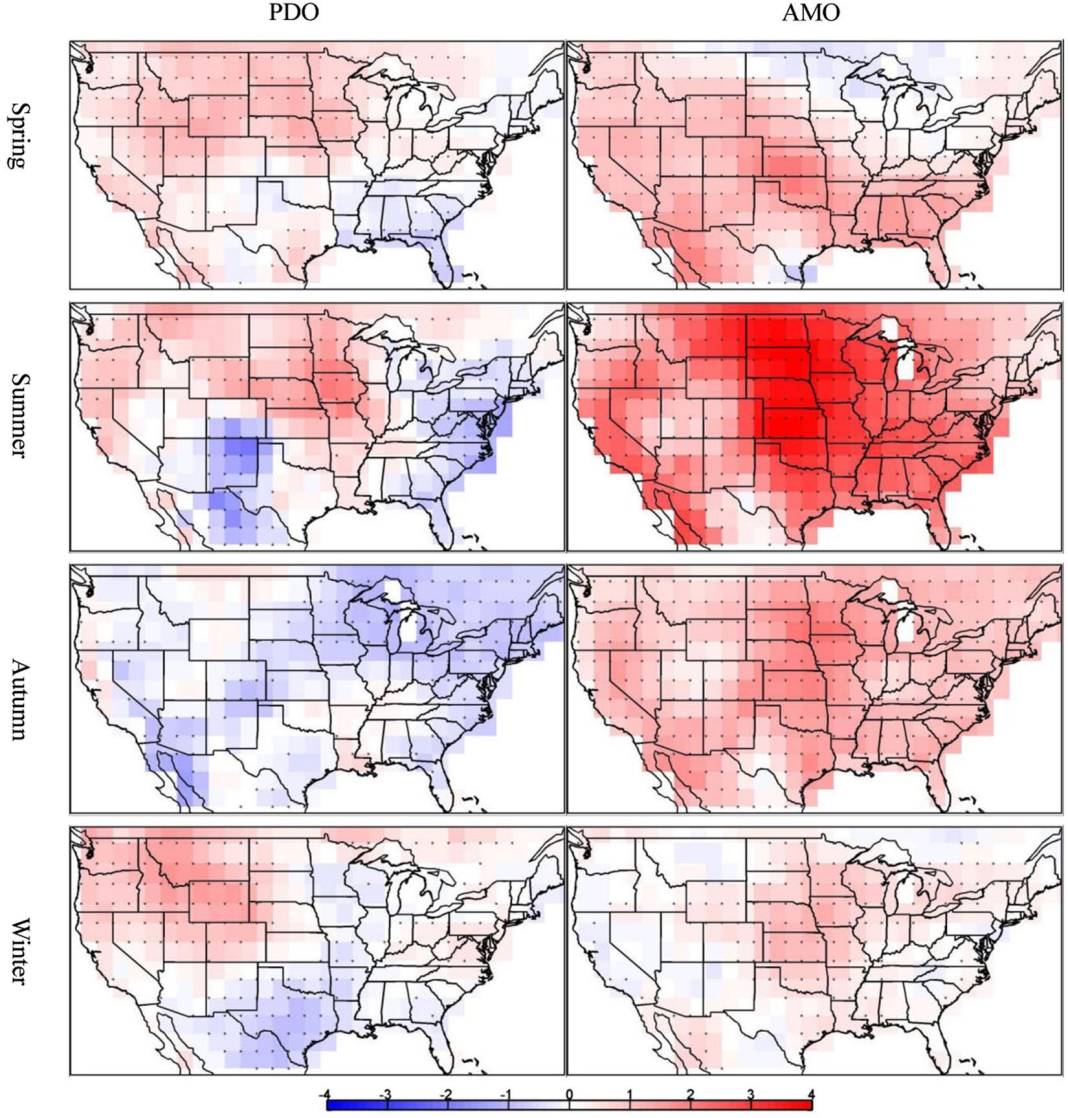
The number of the seasonal warm anomalies from the Twentieth Century Reanalysis regressed onto the PDO and AMO indices (unit: day yr^−1^). The dotted regions indicate above 95% confidence level. This figure is created using MATLAB & Simulink Release 2010b (www.mathworks.com).

The long-term data are also used to examine the impact of the two interdecadal climate modes (PDO and AMO) on the number of seasonal warm anomalies. As shown by the regression results in Fig. 9, during the positive phase of the PDO, warm anomalies are more frequent in the western and central U.S. and less frequent in the eastern and southern U.S. for all seasons except for autumn when a decrease in the seasonal warm anomalies occur in the eastern, central and southwestern U.S. The opposite occurs during the negative phase of the PDO. The AMO exerts a stronger effect on the occurrences of seasonal warm anomalies compared with the PDO. In all but the winter season, the positive phase of the AMO usually results in more frequent occurrences of warm anomalies across the U.S., with the exception of the springtime Great Lakes region. In winter, the region with increased frequency shrinks to only the central U.S. The negative phases of the AMO produce an opposite spatial pattern.

## Conclusion and Discussion

Previous studies suggest that the increasing trends in seasonal warm anomalies in the U.S. can be attributed, in large part, to increases in greenhouse gas concentrations in the lower atmosphere. However, it is not clear to what extent natural climate variability contributes to these trends. In this study, we have evaluated the contributions of the low-frequency climate modes characterized by the AMO and PDO to the increasing trends in the seasonal warm anomalies over the past three decades. The positive trend in the AMO and the negative trend in the PDO are favorable for increasing trends in warm anomalies across large parts of the U.S. during all seasons and decreasing trends in warm anomalies during the winter in portions of the Intermountain West and the western Northern Plains. The anomalous height and wind fields related to the trends in the AMO and PDO indices can explain the trends in the occurrence of seasonal warm anomalies. The associations of trends in seasonal warm anomalies with decadal and multi-decadal variability of the AMO and PDO are further confirmed by a century-long dataset. In recent decades, El Niño Modoki has exhibited an increasing trend in its frequency of occurrence^29^. Our results also suggest an association of La Niña Modoki with changes in the number of spring, and especially winter, warm anomalies (Fig. 5).

These results improve our understanding of the role of interdecadal climate modes (AMO and PDO) in contributing to the trends and variability in the occurrence of mid-latitude warm temperature extremes. This role can complicate the relationship between temperature extremes and well-known higher-frequency climate oscillations such as El Nino Southern Oscillation (ENSO)^30^. Significant biases in seasonal forecasts may occur when not considering the influence of these low-frequency climate modes. The results can also be used to anticipate future extreme warm temperature events, which can help in the development of long-term ecological, social and economic planning strategies that account for changes in the climate system.

Finally, it is worth pointing out that despite the evidence linking the recent trends in the occurrences of seasonal warm extremes in regions of the U.S. to low-frequency modes of climate variability characterized by AMO and PDO, it is unlikely that natural variability alone can account for the long-term trends without considering the contributions from increasing greenhouse gas emissions^14–19^. It is also worth noting that our results are based only on statistical analyses; numerical simulations are necessary to fully investigate the potential teleconnections between SST anomalies and warm temperature extremes in the U.S. and to determine the relative importance of natural climate variability compared to anthropogenic climate changes.

## Methods

The analyses use time series of daily maximum temperatures from two reanalysis datasets: the North American Land Data Assimilation System Phase 2 (NLDAS-2)^22^ and the Twentieth Century Reanalysis^23^. NLDAS is a long-term high-resolution atmosphere and land-surface hydrology dataset produced by driving land surface models with a suite of observational and model reanalysis data^31^ over a domain covering most of the North American continent (25°N–53°N; 125°W–67°W). NLDAS-2 corrected the biases in precipitation and downward shortwave radiation found in NLDAS-1, Phase 1 of the NLDAS^32–34^. The advantage of the NLDAS-2 data lies in its high spatial (1/8th degree) and temporal resolution (three hourly). However, the relatively short time-period coverage of the data (1979 – present) limits its utility for fully assessing low-frequency variability modes. The results from the NLDAS-2 analyses are thus verified using the Twentieth Century Reanalysis that spans over a century (January 1871 to December 2012) but at lower temporal (6-hourly) and spatial (2° × 2°) resolutions. The Twentieth Century Reanalysis was generated by assimilating surface pressure and sea-level pressure observations into an Ensemble Kalman Filter data assimilation system, and the National Centers for Environmental Prediction (NCEP) atmosphere-land model is utilized to produce first-guess fields with sea ice and SST fields as prescribed boundary conditions^23^.

The daily maximum temperature time series from these two datasets are extracted and used to determine the number of days in each of the four seasons when the daily maximum temperature exceeds the 95^th^ climatological percentile for that season. The number of these seasonal warm anomalies is examined for the existence of any significant trends and their potential connection to the large-scale, low-frequency modes of climate variability.

The major modes of climate variability have been quantified through various indices. For this study, we used the monthly Pacific Decadal Oscillation (PDO) index^24^ available from the University of Washington (http://www.atmos.washington.edu/~mantua/abst.PDO.html) and the monthly Atlantic Multi-decadal Oscillation (AMO) index^25^ available from the National Oceanic and Atmospheric Administration (NOAA) http://www.esrl.noaa.gov/psd/data/correlation/amon.us.data). In addition, large-scale atmospheric circulations corresponding to the seasonal warm anomalies in the NLADS-2 dataset were derived from the NCEP-Department of Energy (DOE) global reanalysis dataset^35–37^, which has a horizontal resolution of 2.5° latitude × 2.5° longitude and a temporal resolution of 6 hours. The NOAA Extended Reconstructed SST dataset^38,39^ was used for identifying the SST patterns over the North Pacific and Atlantic Oceans.

Empirical-orthogonal-function (EOF) analyses were utilized to identify the dominant patterns in the number of seasonal anomalous maximum temperature occurrences relative to the climatology of the study period in the two datasets (1979–2014 for NLDAS2 and 1871–2012 for the Twentieth Century Reanalysis). The relationships to the large-scale climate modes were established via regression analyses.

For the analyses utilizing the Twentieth Century Reanalysis data, since the focus is on decadal and multi-decadal time scales, the original time series was detrended, and a 20-year low-pass filter was applied to eliminate high frequency variability. The AMO and PDO time series for the same period were similarly detrended and filtered. Similar to the short-term data, an EOF analysis was applied to the anomalies of seasonal warm anomalies.

The gridded reanalysis data may underestimate the influence of local factors such as terrain and vegetation on daily maximum temperatures, but the limitation is unlikely to influence the confidence in the results of the current analyses. The gridded reanalysis datasets are also known to underestimate extreme values due to relatively low spatial and temporal resolution. They are, however, adequate in capturing the variability of the extreme values. Mutiibwa *et al*.^40^ estimated spatiotemporal patterns of temperature extremes across conterminous United States in the recent decades using NLDAS-2 data. To determine how well the two reanalysis datasets capture the variability, we compared the surface daily maximum temperatures at three climate stations in Michigan with the daily maximum temperatures derived from the NLDAS-2 and 20th century reanalysis data and the results are shown for the year 2010 (Fig. S1). As expected, there exists a cold bias in both reanalysis time series, ranging from 0.92–2.85 °C for NLADS-2 to 3.11–3.89 °C for the 20^th^ century reanalysis. The results for the other years (not shown) are similar. The cold bias is important to consider when assessing a specific warm event or a series of events because the strengths of the event(s) would be underestimated. However, as shown in Fig. S1, the variability in the observed time series is captured reasonably well by both reanalysis datasets, with correlation coefficients of 0.59, 0.74, and 0.81 for Maple City, Hart and Eau Claire for NLDAS-2, and 0.67, 0.70, and 0.61, respectively for the three sites for the 20th century reanalysis. Also revealed by Fig. S1 are some significant seasonal differences in the magnitude of the cold bias, which can have an effect on the anomalies being studied.

## Electronic supplementary material

Figure S1
