# Supplementary material for: Trends in seasonal warm anomalies across the contiguous United States: Contributions from natural climate variability
Source: Sci Rep. 2018 Feb 21;8:3435. doi: 10.1038/s41598-018-21817-9 (PMC5821865; doi:10.1038/s41598-018-21817-9)
Supplement: Supplementary file 1 — Figure S1 [file 41598_2018_21817_MOESM1_ESM.pdf]

Trends in seasonal warm anomalies across the contiguous United States: Contributions from natural climate variability

Lejiang Yu[1,2*], Shiyuan Zhong[2*], Warren E. Heilman[3], Xindi Bian[3]

[1]SOA Key Laboratory for Polar Science, Polar Research Institute of China, Shanghai, China

[2]Department of Geography, Environment and Spatial Sciences, Michigan State University, East Lansing, MI, USA

[3]Northern Research Station, US Forest Service, Lansing, Michigan, USA

*Corresponding Authors' address

Dr. Lejiang Yu

SOA Key Laboratory for Polar Science, Polar Research Institute of China, Shanghai, China

Jinqiao Road 451, 200136, Shanghai, China

Phone: 0086-020-58712034, email: yulejiang@sina.com.cn

Dr. Shiyuan Zhong

Department of Geography, Environment and Spatial Sciences, Michigan State University, East Lansing, MI, USA

674 Auditorium Rd., East Lansing, MI, 48824, USA

Phone: 001-517-432-4743, email: zhongs@msu.edu

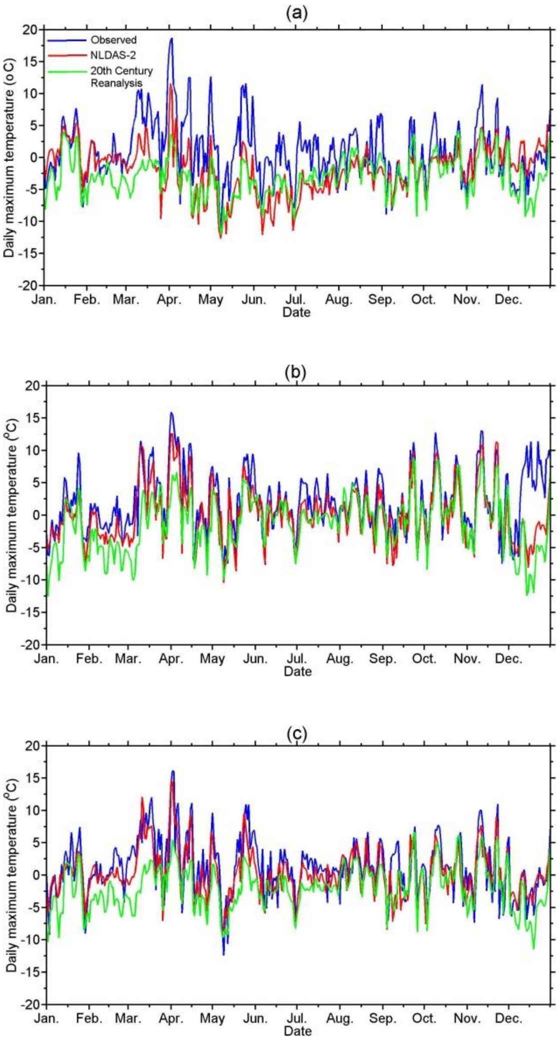

Figure S1 Comparison of surface daily maximum temperature of the NLDAS2 data (red) and the Twentieth century reanalysis data (green) with the observations (blue) collected at (a) Maple City (44.85 °N, 85.85W), (b) Eau Claire (42.02 °N, 86.25W) and (c) Hart (43.70 °N, 86.37W) in the state of Michigan for the year 2010. The seasonal cycle has been

removed before plotting. This figure is created using MATLAB & Simulink Release 2010b (www. mathworks.com).
